# Editorial: Health of adolescents: Quantitative and qualitative perspective

**DOI:** 10.3389/fpsyg.2023.1156334

**Published:** 2023-03-22

**Authors:** Jaroslava Kopcakova, Daniela Husarova, Erik Sigmund, Gabriel Banik, Lenka Sokolova

**Affiliations:** ^1^Department of Health Psychology and Research Methodology, Faculty of Medicine, University of Pavol Jozef Šafárik in Kosice, Kosice, Slovakia; ^2^Faculty of Physical Culture, Palacký University Olomouc, Olomouc, Czechia; ^3^Institute of Psychology, Faculty of Arts, University of Presov, Presov, Slovakia; ^4^Institute of Applied Psychology, Faculty of Social and Economic Sciences, Comenius University, Bratislava, Slovakia

**Keywords:** health, adolescents, quantitative research, qualitative research, mental health

The current generation of adolescents differs from previous generations, because the development of new technologies and their use in ever-younger children is an unprecedented formational influence, especially during the Covid pandemic. Based on trends in international research, quantitative and qualitative participatory approach seems to be the most important source of information about health and health-related behavior of the current adolescent generation.

This Research Topic aimed to highlight the health of adolescents from the quantitative and qualitative perspectives of research. Adolescence is a period of extensive psychological changes, such as the need to explore, growing independence, developing self-concept and the need for peer acceptance and family support. The benefits of an active childhood can carry over into adulthood, therefore the establishment of healthy patterns during childhood and adolescence plays important role. Unfortunatelly, the current generation of adolescents has to face several socio-economic challenges and crises (the Covid pandemic, the war conflict in Ukraine, the energy crisis, the climate crisis, etc.), which have a significant impact on their mental health. Research showed, that adolescents often experience feelings of helplessness, insecurity, isolation, insignificance or loneliness (Šinanská, 2019). Moreover, approximately 10–20% of children and adolescents suffer from emotional and behavioral problems (Jaspers et al., 2012), what is also confirmed by the findings of the Health Behavior in School-Aged Children Study (Lacková Rebičová et al., 2019; Inchley et al., 2020). During the Covid pandemic, there was an even larger increase in adolescent mental health problems such as depression (Ma et al., 2021; Thorisdottir et al., 2021), hyperactivity, behavioral problems (Waite et al., 2021), anxiety and sleep problems (Ma et al., 2021). Previous research also suggests that older adolescents may suffer a decline in mental health outcomes, with older girls reporting poorer mental health than older boys (Inchley et al., 2020). This evidence has led to growing awareness of the need to address adolescents' mental health requirements by identifying the factors that can promote or hinder their mental health in a different setting.

Mental health, like other aspects of health, can be affected by a range of factors that need to be addressed through comprehensive strategies of support, prevention and treatment. Determinants of mental health and mental disorders do not include individual attributes alone, but social, cultural, economic, political and environmental factors (Currie and Morgan, 2020; World Health Organization, 2021) as well, which are covered in Bronfenbrenner's ecological model (Bronfenbrenner, 1992). At the individual level, these are factors such as gender, age, level of physical activity, length, and quality of sleep, risk behavior, presence of chronic disease and medication use (Levin et al., 2009; Gobina et al., 2011; Santos et al., 2015; Kleszczewska et al., 2018; Evans et al., 2019). In the context of social factors, the environment in which adolescents live and spend time, including relationships with peers, classmates, and teachers, plays a significant role (Currie and Morgan, 2020). These factors influence each other and interact at different levels within the family, school and peers (Moore et al., 2018). Understanding the impact of risk factors on mental health is essential to mitigating the impact of the various negative events of the present time.For this reason, the topic of mental health has become part of the action plan of the World Health Organization, whose vision is a world in which mental health is valued, supported and protected (World Health Organization, 2021).

In the current Research Topic, we covered a wide range of mental health issues among adolescents. A study by Lackova Rebicova et al. focused on adverse childhood experiences which can cause serious mental problems in adolescents and therefore may be expected to be associated with higher use of psychosocial care, potentially varying by type of specific adverse childhood experience. The study found that having three or more adverse childhood experiences as well as experiencing some specific adverse childhood experiences (death of a mother/father, death of somebody else you love, problems of a parent with alcohol or drugs, conflicts or physical fights between parents, and separation/divorce of parents) increased the likelihood of using psychosocial care.

Lukoševičiute et al. studied the happiness of adolescents in three European regions and they concluded that health complaints, bullying behaviors, and self-directed violence were related to lower levels of happiness. Macek et al. reported relatively stable levels of life satisfaction, self-esteem, and self-reported daily hassles in adolescents in the period from 1992 to 2019. The above-mentioned findings showed a negative impact on the mental health and wellbeing of adolescents.

Another study by Jozefiakova et al. studied Covid anxiety and its predictors among Slovak adolescents. Based on the results from this quantitative study, lower resilience, higher attachment anxiety, being a girl and having a higher age are predictors related to higher Covid anxiety. Similarly, the impact of the COVID lockdown was more negative in girls in the study of Furstova et al.. Based on these findings, the Covid pandemic seems to be harmful to adolescent mental health and girls are at higher risk.

Modern technology plays a significant role in the life of adolescents, but public perception and policy are mostly dominated by their perceiving as disadvantages and risks. The results of online semi-structured interviews with Slovak adolescents showed that adolescents perceived modern technology as the most supportive and helpful tool in their life (Bitto Urbanova et al.).

Even though digital technology is an important part of adolescents' everyday routine, social interactions with significant others play a key role, as well as the home environment and family climate. Adolescence is often connected with the occurrence of different types of risk behaviors. Qing et al. who studied sibling bullying showed, that 29% of Chinese adolescents in their sample were involved in this type of risk behavior. Furthermore, according to Jiang et al., deviant behavior is positively affected by academic pressure in Chinese adolescents.

The studies included in this Research Topic reflected several important issues in the health of adolescents. Eperiences of global threats like the Covid pandemic together with challenges including digital and social life affect the wellbeing and health of adolescents and therefore, the focus on adolescent health research is still very important and essential.

## Author contributions

All authors listed have made a substantial, direct, and intellectual contribution to the work and approved it for publication.

## References

[B1] Bronfenbrenner U. (1992). Ecological Systems Theory. London: Jessica Kingsley Publishers.

[B2] Currie C. Morgan A. A. (2020). bio-ecological framing of evidence on the determinants of adolescent mental health - A scoping review of the international Health Behaviour in School-Aged Children (HBSC) study 1983-2020. SSM Populat. Health. 12, 100697. 10.1016/j.ssmph.2020.100697 33335971 PMC7732871

[B3] Evans D. S. O'Farrell A. Sheridan A. Kavanagh P. (2019). Comparison of the health and well-being of smoking and non-smoking school-aged children in Ireland. Child. 45, 694–701. 10.1111/cch.12681 31039602

[B4] Gobina I. Välimaa R. Tynjäl,ä J. Villberg J. Villerusa A. Iannotti R. J.. (2011). The medicine use and corresponding subjective health complaints among adolescents, a cross-national survey. Pharmacoepidemiol. Drug Safety. 20, 424–431. 10.1002/pds.2102 21246643

[B5] Inchley J. Currie D. Budisavljevic S. Torsheim T. Jåstad A. Cosma A.. (2020). Spotlight on Adolescent Health and Well-Being. Findings from the 2017/2018 Health Behavior in School-aged Children (HBSC) survey in Europe and Canada. International report. Vol. 1. Key findings. Copenhagen: WHO Regional Office for Europe.

[B6] Jaspers M. de Winter A. F. Huisman M. Verhulst F. C. Ormel J. Stewart R. E.. (2012). Trajectories of psychosocial problems in adolescents predicted by findings from early well-child assessments. J. Adolesc. Health 51, 475–483. 10.1016/j.jadohealth.2012.02.007 23084169

[B7] Kleszczewska D. Dzielska A. Salonna F. Mazur J. (2018). The association between physical activity and general life satisfaction in lower secondary school students: The role of individual and family factors. Commun. Mental Health J. 54, 1245–1252. 10.1007/s10597-018-0309-x PMC6208981 30076505

[B8] Lacková Rebičová M. Dankulincová Veselská Z. Husárová D. Madarasová Gecková A. van Dijk J. P. Reijneveld S. A.. (2019). The number of childhood experiences is associated with emotional and behavioural problems among adolescents. Int. J. Environ. Res. Public Health 16, 2446. 31324072 10.3390/ijerph16132446 PMC6651534

[B9] Levin K. A. Currie C. Muldoon J. (2009). Mental well-being and subjective health of 11-to 15-year-old boys and girls in scotland, 1994–2006. Eur. J. Public Health. 19, 605–610. 10.1093/eurpub/ckp046 19383842

[B10] Ma L. Mazidi M. Li K. Li Y. Chen S. Kirwan R.. (2021). Prevalence of mental health problems among children and adolescents during the COVID-19 pandemic: A systematic review and metaanalysis. J. Affect. Disor. 293, 78–89. 10.1016/j.jad.2021.06.021 34174475 PMC9711885

[B11] Moore G. F. Cox R. Evans R. E. Hallingberg B. Hawkins J. Littlecott H. J.. (2018). School, peer and family relationships and adolescent substance use, subjective wellbeing and mental health symptoms in wales: A cross sectional study. Child Indic. Res. 11, 1951–1965. 10.1007/s12187-017-9524-1 30524519 PMC6244918

[B12] Santos T. de Matos M. G. Simões C. Machado M. d. C. (2015). Psychological well-being and chronic condition in Portuguese adolescents. Int. J. Adolesc. Youth. 20, 334–345. 10.1080/02673843.2015.1007880

[B13] Šinanská K. (2019). The loneliness of adolescents from socially disadvantaged background. J. Interdiscip. Res. 9, 284–286.

[B14] Thorisdottir I. E. Asgeirsdottir B. B. Kristjansson A. L. Valdimarsdottir H. B. Tolgyes E. M. J. Sigfusson J.. (2021). Depressive symptoms, mental wellbeing, and substance use among adolescents before and during the COVID-19 pandemic in Iceland: a longitudinal, populationbased study. Lancet Psychiat. 8, 663–672. 10.1016/S2215-0366(21)00156-5 34090582

[B15] Waite P. Pearcey S. Shum A. Raw J. A. L. Patalay P. (2021). and Creswell, C. How did the mental health symptoms of children and adolescents change over early lockdown during the COVID-19 pandemic in the UK? JCPP Adv. 1, e12009. 10.1111/jcv2.12009 34485988 PMC8206715

[B16] World Health Organization. (2021). Comprehensive mental health action plan 2013–2030. World Health Organization.License: CC BY-NC-SA 3, 0. IGO Available online at: https://apps.who.int/iris/handle/10665/345301 (accessed January 31. 2023).

